# Life after conflict-related amputation trauma: a clinical study from the Gaza Strip

**DOI:** 10.1186/s12914-018-0173-3

**Published:** 2018-08-31

**Authors:** Hanne Edøy Heszlein-Lossius, Yahya Al-Borno, Samar Shaqqoura, Nashwa Skaik, Lasse Melvaer Giil, Mads Gilbert

**Affiliations:** 1 The Anaesthesia and Critical Care Research Group, Institute of Clinical Medicine, The Arctic University of Norway, Tromsø, Norway; 2 Al-Shifa Medical Centre, Gaza Strip, Gaza, Palestine; 3 Department of Internal Medicine, Haraldsplass Deaconess Hospital, Bergen, Norway; 4 Clinic of Emergency Medicine, University Hospital of North Norway, Tromsø, Norway; 5 UiT The Arctic University of Norway, Tromsø, Norway

**Keywords:** Amputees, Gaza, Israel, Military incursion, Modern warfare, Palestine, Trauma

## Abstract

**Background:**

More than 17.000 Palestinians were injured during different Israeli military incursions on the Gaza Strip from 2006 to 2014. Many suffered traumatic extremity amputations. We describe the injuries, complications, living conditions and health among a selection of traumatic amputees in the Gaza Strip.

**Methods:**

We included 254 civilian Palestinians who had survived, but lost one or more limb(s) during military incursions from 2006 to 2016. All patients were receiving follow-up treatment at a physical rehabilitation center in Gaza at the time of inclusion. We measured and photographed anatomical location and length of extremity amputations and interviewed the amputees using standard questionnaires on self-reported health, socioeconomic status, mechanism of injury, physical status and medical history.

**Results:**

The amputees were young (median age 25,6 years at the time of trauma), well educated (37% above graduate level), males (92%), but also 43 children (17% ≤ 18 years). The greater part suffered major amputations (85% above wrist or ankle). Limb losses were unilateral (35% above-, 29·5% below knee), and bilateral (17%) lower extremity amputations. Pain was the most frequent long-term complaint (in joints; 34%, back; 33% or phantom pain; 40·6%). Sixty-three percent of amputees were their family’s sole breadwinner, 75·2% were unemployed and 46% had lost their home. Only one in ten (11·6%) of the destroyed homes had been rebuilt.

**Conclusions:**

The most frequently observed amputees in our study were young, well-educated male breadwinners and almost one in five were children. Conflict-related traumatic amputations have wide-ranging, serious consequences for the amputees and their families.

## Background

During four major Israeli military incursions (Operation Summer Rain 2006, Operation Cast Lead 2008–09, Operation Pillar of Defense 2012, Operation Protective Edge 2014), around 4000 Palestinians were killed and over 17.000 injured (2006: 412 killed/ 1264 injured, 2009: 1383 killed/> 5300 injured/, 2012: 130 killed/1399 injured, 2014: 2251 killed/11231 injured) [1–3]. Many survivors lost one or more limb(s). Mechanisms and severity of amputation injuries as well as the living conditions and health of the amputees in Gaza has to our knowledge not been systematically studied. The literature on conflict related trauma is mainly focused on military personnel, not on civilians, making comparison with previous studies difficult. More in depth studies on the civilians in Gaza with conflict related amputations has been called upon by health professionals [4]. Non-peer reviewed articles and human right groups reports on the civilian consequences of conflict related amputations are available, many of them from war-torn areas in Afghanistan and Pakistan. In Afghanistan the country’s growing number of amputees face harsh living conditions with financial and social deterioration [5].

Studies conducted on military personnel has provided knowledge on the long-term medical complications from conflict related amputations occurring in combat.

In a cross-sectional study on traumatic amputee veterans from the Vietnam war and Operation Iraqi Freedom, the most frequently reported long-term complications were chronic back pain (36.2% and 42·1%), phantom pain (72·2% and 76%), and residual-limb pain (48·3% and 62·9%) [6]. In a retrospective study on traumatic amputations, the amputees were found to have a near doubled risk of infections, septicemia, anemia, and thromboembolic disease compared to those with extremity injuries without amputation [7]. Poor mental health is prevalent among amputees with low education [8]. Chronic pain syndromes are common among civilian victims of traumatic extremity amputations caused by landmines [9]. The strain placed on the Gazan healthcare system from repetitious military attacks has in previous studies been highlighted as severe [10]. Gazans require referral permits to access more advanced medical care outside of Gaza, and reports from human rights observers are indicating that the approval of such referrals are hard to obtain and in general decreasing [11]. Complicated access to adequate treatment may hamper possibilities for healing and recovery with further burdens for the amputees. We hypothesize that the personal losses of amputees are reaching beyond the sheer loss of limb(s).

The aim of our study was to describe extent and consequences of traumatic amputations among Palestinians in the Gaza Strip attending rehabilitation. We recorded mechanisms of injury, severity of amputations, post-amputation complications, living conditions, and psychosocial trauma in a population of civilian amputees followed up in the main rehabilitation center in Gaza, The Artificial Limb and Polio Centre in Gaza City (ALPC).

## Methods

### Study participants

We included Palestinians in Gaza who had one or more traumatic amputation(s) caused by a weapon in the time frame 2006–2016. This period was chosen because it includes four major military incursions. The cross-sectional study was conducted from June 2014 to December 2016. Among 1170 patient records screened at ALPC, 254 patients met our inclusion criteria, while 915 were excluded because amputations were not war related or happened prior to 2006 (Fig. 2).

This study followed a pilot study of 90 patients (June-Nov. 2014) from the ALPCs patient register who were the first 90 found to meet the inclusion criteria. The 90 patients were invited to participate by one phone call made by a health secretary at the ALPC. The response rate in the pilot group was 99%. Following the pilot study, we proceeded to invite all patients from the ALPC register who met the inclusion criteria. ALPC is the only producer and provider of artificial limbs in Gaza. The center offers good physical facilities patients could be examined and interviewed, running water and stable power supply thanks to well-functioning generators supplying electricity during the daily power outages caused by the on-going siege of Gaza. The ability and permission to have the ALPC as the study center had significant security advantages. Here, researchers could meet the patients in a set and relatively safe place. Also, this avoided travels for researcher’s home visits in situations when there was ongoing attacks or incursions. All services offered by ALPC to the amputees in Gaza is free of charge and access to treatment was independent of the patient’s financial status.

An experienced physician examined each patient and recorded his or her physical status and medical information. The patients were given printed questionnaires in Arabic, designed for yes/no answers or for Likert-scale graded answers. The questionnaires assessed the mechanism of injuries, socioeconomic status, amputation-related complications, co-morbidity, use of artificial limbs, and ongoing therapy. The questions were quality assured by translation-retranslation between English and Arabic. Two well validated forms were used: the 12-question General Health Quality survey (GHQ-12) and Short Form Health survey (SF-36) [12, 13]. The questionnaire assessing amputation-related complications was inspired by the questionnaire made by Reiber, McFarland, Hubbard, et al. in their study *Servicemembers and veterans with major traumatic limb loss from Vietnam war and OIF/OEF conflicts: survey methods, participants, and summary findings*.^1^

**Fig. 1:**
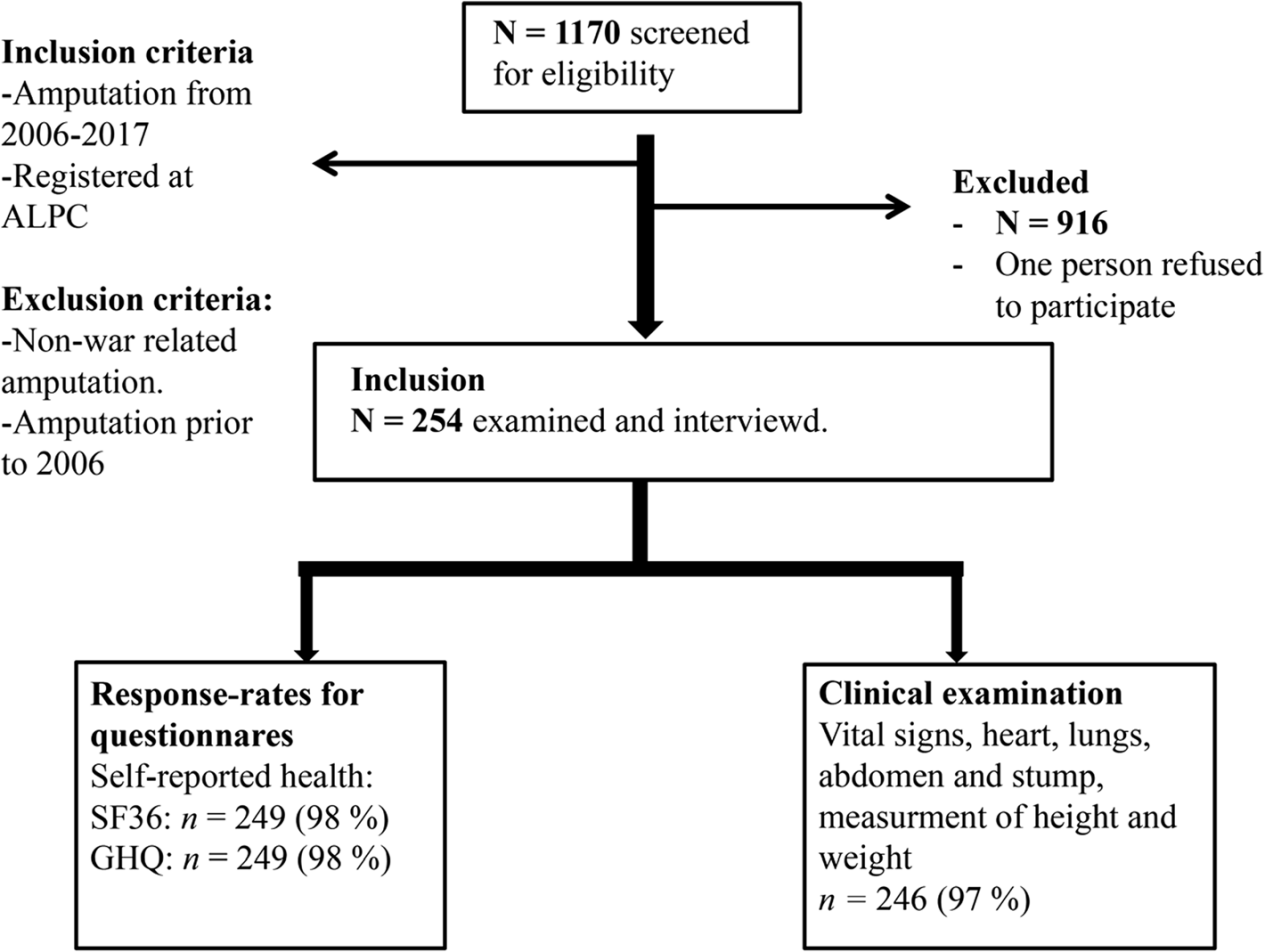
Inclusion of study participants. *N = 1170. ALPC: Artificial Limb and Polio Center, Gaza’s main rehabilitation clinic where patients were selected for participation

The patients completed the questionnaires prior to meeting with the study physicians. One of the investigators read the questions out loud when examining illiterate patients. The procedures for study inclusion are summarized in Fig. 1.

### Ethics, consent and permission

The ALPC location was chosen as the local study base in accordance with the local health authorities, the board of Gaza’s main hospital, Al-Shifa Hospital (Gaza’s trauma center), and the ALPC director. All patient completed written consent prior to participating. The study was approved by the Regional Ethical Committee (approval number: 2016/1265/REK Nord) in Norway and the Committee for Helsinki ethics approvals in Gaza. All participants’ roundtrip travel expenses to ALPC were reimbursed.

### Clinical examination

A standardized clinical examination was performed with measurement of heart rate, blood pressure, height and weight as well as auscultation of heart, lungs and examination of the abdomen. The amputation stump(s) were examined for ulcerations, pain, and tumors. Each stump was photographed and photos labelled with a unique patient number.

### The questionnaires

#### The 12-item general health questionnaire (GHQ-12)

The GHQ-12 is a 12-questions screening tool commonly used to detect mental illness in the general population in a community. It is self-administrated and easy to complete. The Arabic version has been validated for use in Arab-speaking patients and used to map occupational stress among hospital nurses in Gaza [13, 14].

#### The thirty-six item short form survey (SF-36)

The SF-36 is a multi-purpose, short-form self-administered questionnaire with 36 questions. Items are organized to give an eight-scale profile-score of health and well-being [12]. The results are presented with one summary of physical components and one with mental health components.

#### Socioeconomic status

Socioeconomic status was assessed by asking the participants about their level of education, family situation, number of persons in the household, the number of siblings, employment, perceived reasons for unemployment, income, family income, and the number of dependents. In addition, the destruction and reconstruction of the patients’ homes were recorded.

#### Personal loss

Twelve patients had been interviewed and examined prior to the start of the military incursion “Operation Protective Edge” July–August 2014. Patients included after this were asked about their specific experiences during this period. Patients spoke freely with the examining medical doctor about their personal losses and loss of spouse, children, other family members and/or friends.

### Mechanisms of injuries

Each patient was asked to report on types and modes of weapon or explosive they knew or believed to have caused the injury leading to the amputation. The patients told the interviewer about witnesses, hospital reports and their own knowledge of various weapons used. The Palestinian residents of Gaza have experienced multiple, recurrent military attacks, and are used to differentiate between different weapons and weapon carriers. The various weapon delivering systems (attack helicopters, fighter jets, naval artillery, tank artillery, drones etc.) and their potential for trauma will be subjects in later publications.

### Level of amputation

The level of each extremity amputations were examined and classified as above or below the concomitant extremity joint.

Major amputations were defined as limb amputations above wrist or ankle. Minor amputations were defined as amputations below wrist or ankle. The examining physician recorded amputation levels by drawing on an anatomical sketch for each patient.

### Statistics

Descriptive statistics are reported as mean and standard deviation (SD) for parametric data, median and interquartile range (IQR) for non-parametric data. We consider a *p*-value < 0·05 statistically significant. Frequencies are reported as percentage of the total study population for groups and subgroups. Alluvial flow diagrams are used to visualize complex relations between categorical variables. We assessed medical complications by multiple correspondence analysis (MCA) with principal normalization. In MCA, one seeks to identify the relationships between the measured matrices and potential latent variables. MCA uses the contingency tables as the matrix of relation and answers which of the multiple complications that are related to each other. Data analysis is conducted in SPSS Statistics version 22.0 (SPSS Inc., Chicago, IL, USA) and STATA 15 (StataCorp. 2015. *Stata Statistical Software: Release 15*. College Station, TX: StataCorp LP), with graphical displays from RAW Graphs (https://rawgraphs.io/about/).

## Results

### Patient demographics and war-related loss

The study-population included 254 Palestinian patients with traumatic extremity amputations residing in Gaza. All had rehabilitation treatment with fitting of artificial limb(s), physiotherapy and training at the ALPC. Patient characteristics are described in Table 1.

**Table 1:** Characteristics of study participants^a^ (*N* = 254)

Variables	n (patients)	Percentage or median, [IQR]
Demographics^b^		
Palestinian	254	100
Male	234	92
Children	43	17
Female	20	8
Refugee status	154	57
Age –Inclusion, years		28 [10]
Age-Injury, years		23 [9]
Education^c^		
Illiterate	9	4
Elementary school	45	18
Secondary school	75	30
High school	26	10
Graduate	22	9
Postgraduate	70	28
PhD	1	0·4
Destruction of home^d^		
Loss of home	116	46
Treatment^e^		
Uses artificial limb	142	56
Waiting for artificial limb	38	15
Not using	72	28
Receives physiotherapy	215	85
Financial situation^f^		
Household, Hamula	103	41
> 8 Persons per household	135	54
> 6 Siblings	185	74
Family income, NIS		
< 700	76	30
800–1600	105	42
> 1700	50	28

*Abbreviations:* *IQR* interquartile range

^a^Number of participants: 254, from 0 to 2% of the participants had missing data on any variable

^b^Refugee = patient is from a family who have a refugee status as of 1948

^c^Secondary school = 12 years of education, Graduate = has completed the first academic degree in university, post-graduate = completed Master degree

^d^Number of patients who lost their home in one of the incursions

^e^Treatment 38 patients were at the time of inclusion waiting for an artificial limb to be fitted due to recent trauma. 72 patients did not use their fitted artificial limbs due to different reasons

^f^Financial situation: Hamula: The Palestinian term for extended family. Here compared with the nuclear family

*NIS* New Israeli Shekel. 1 NIS equals 0,26 US Dollar

Twenty-four patients were amputated during the military incursion, *Operation Summer Rain*, in 2006, 57 patients were amputated during *Operation Cast Lead* in 2008/90, 4 patients were amputated during *Operation Pillar of Defense,* in 2012 and 73 patients were amputated during the latest military incursion, *Operation Protective Edge,* in 2014. Ninety-five patients were amputated between these periods of declared military operations and one person only provided the year, but no exact date of the injury leading to amputation.

Most amputees were males in their early 20s when injured and in their late 20s at inclusion. Seventeen percent were amputated when they were children (aged 18 years or younger, *n* = 43) (Table 1). The majority were well educated. Illiteracy rate was low. The overall unemployment rate was 75% (*n* = 191) and 44% (*n* = 112) had lost their jobs as a result of the traumatic amputation(s). Almost half of the amputees lived in extended families with large households. Nearly half of the amputees (46%, *n* = 116) had lost their home in one of the attacks. Few destroyed homes had been rebuilt (11·6%, *n* = 29). In the subgroup of amputees asked about loss of family and friends during the incursion in 2014 *(n* = 242), 13% reported loss of at least one family member, while nine amputees (4%) had lost one or more children (Table 2).

**Table 2:** Personal losses during the 2014 incursions

Loss of:	n patients	Percentage
≥ 1 children	9	4
Spouse	3	1
Friend	22	9
Parent	8	3
≥ 1 Sibling	12	5
Total	54	22

*Abbreviations: N* = number (of participants), *N* = 242 participants

Children: Four patients lost one child, two patients lost two children, two patients lost three children and one patient lost four children

Siblings: Seven patients lost one sibling, three patients lost two siblings, one patient lost three siblings and one patient lost four siblings

Thirty per cent of the amputees (76/254) were surviving on less than 700 New Israeli Shekel (100 NIS = 32 USD) monthly and 63% (160/254) were the sole breadwinner for more than three persons in their household. (Table 3).

**Table 3:** Employment status

Variables	n Patients	Percentage
Employment^a^		
Unemployed	191	75
Unemployed due to amputation	112	44
≥ 3 unemployed family members	152	61
≥ 3 persons economically dependent on amputee	160	64

Number of participants: 254, from 0 to 2% of the participants had missing data on any variable

^a^Employment: ≥ 3 persons economically dependent on amputee- the amputee is the solve breadwinner for more than three persons in his/her household

### Classification and anatomical localization of amputations

**Fig. 2:**
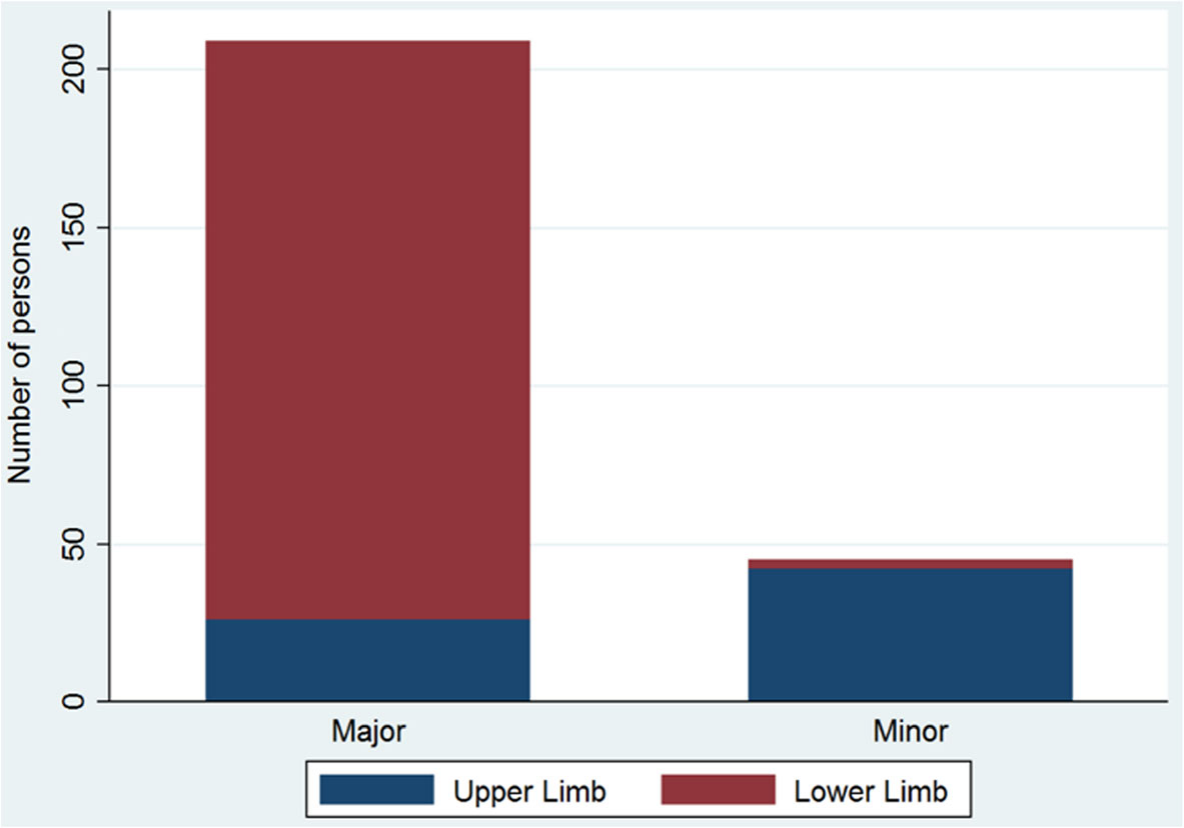
Minor and major amputations by limbs (*N* = 254). * *N* = 254. Nearly nine out of ten amputations were major amputations: 216 (85%) suffered amputations proximal to wrist or ankle. Major = proximal to wrist or ankle. Minor = distal to wrist or ankle

**Fig. 3:**
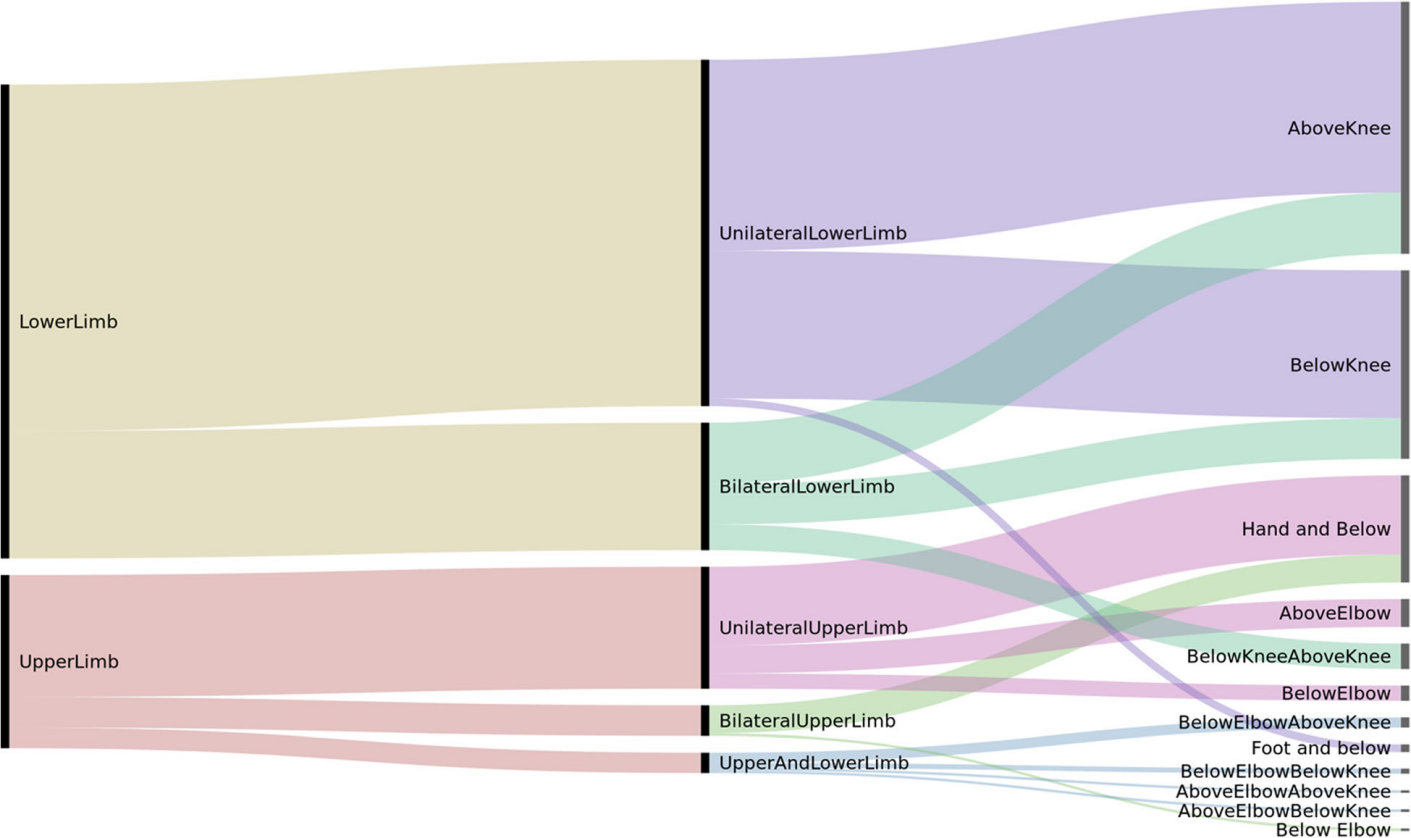
Frequency of amputations by classification and anatomical location (*N* = 254). *N = 254. The alluvial flow diagram shows how the general category (lower limb and upper limb left side) contains parts of finer classification, moving towards the right. The bold vertical lines indicate relative frequency. The colour code shows how each category moves into sub-categories, but change colour in the next step

Nearly nine out of ten of patients had major amputations (*n* = 216, 85%). Lower limb amputations were the most common major and most minor amputations were in the upper extremities (Fig. 2). As illustrated in the Alluvial flow diagram (Fig. 3), unilateral above knee amputations were the most frequent amputation among the patients (*n* = 89, 35%). Bilateral amputations were most often found above the knees (*n* = 27, 11%), while bilateral amputations below the knees occurred in 7% (*n* = 17). Among patients with upper limb amputations, the most common amputation was distally in the arm and hand. Twenty-one patients (8%) had both upper and lower limb amputations.

### Rehabilitation and use of artificial limbs

The 254 patients included in this study increased the caseload on the rehabilitation center by 28%. More than half of the amputees were using an individually fitted artificial limb (142/254, 56%), while 38 (15%) of the patients were waiting for their prosthesis. Seventy-two patients (28%) were not using their prosthesis; five (2%) because it was painful, 39 (15%) claimed their functions were better without prosthesis and eight patients (3%) had an injury judged unsuitable for prosthesis. Twenty-one (8%) patients avoided using their artificial limb because they ‘disliked it’. Amputees with below knee amputations were more often using their prosthesis compared to those with above knee amputations. Among above-knee amputees, 59% (*n* = 52) were using prosthesis compared to 72% (*n* = 54) of those with below-knee amputations.

Rehabilitation physiotherapy was given to 215 (85%) of the patients at a regular basis, while 33 (13%) had not started the training at the time of the study.

### Surgeries after the injury

Most amputees underwent several surgical interventions after the initial amputation trauma, and 53/254 (21%) of the amputees had more than 10 surgical operations, while 91/254 (36%) had at least 4 surgeries after their initial trauma (Table 4). Around 1300 surgical operations were needed to treat the surgical complications and adjust the amputation stumps among the 254 amputees.

**Table 4:** Post-injury surgery

Operations	n patients	Percentage
0-1	44	17
2-3	63	25
4-5	47	19
6-7	21	8
8-9	23	9
>10	53	21

Post-injury surgery: Number of operations performed after trauma

Number of participants: 254, from 0 to 2% of the participants had missing data on any variable

### Medical problems

**Fig. 4:**
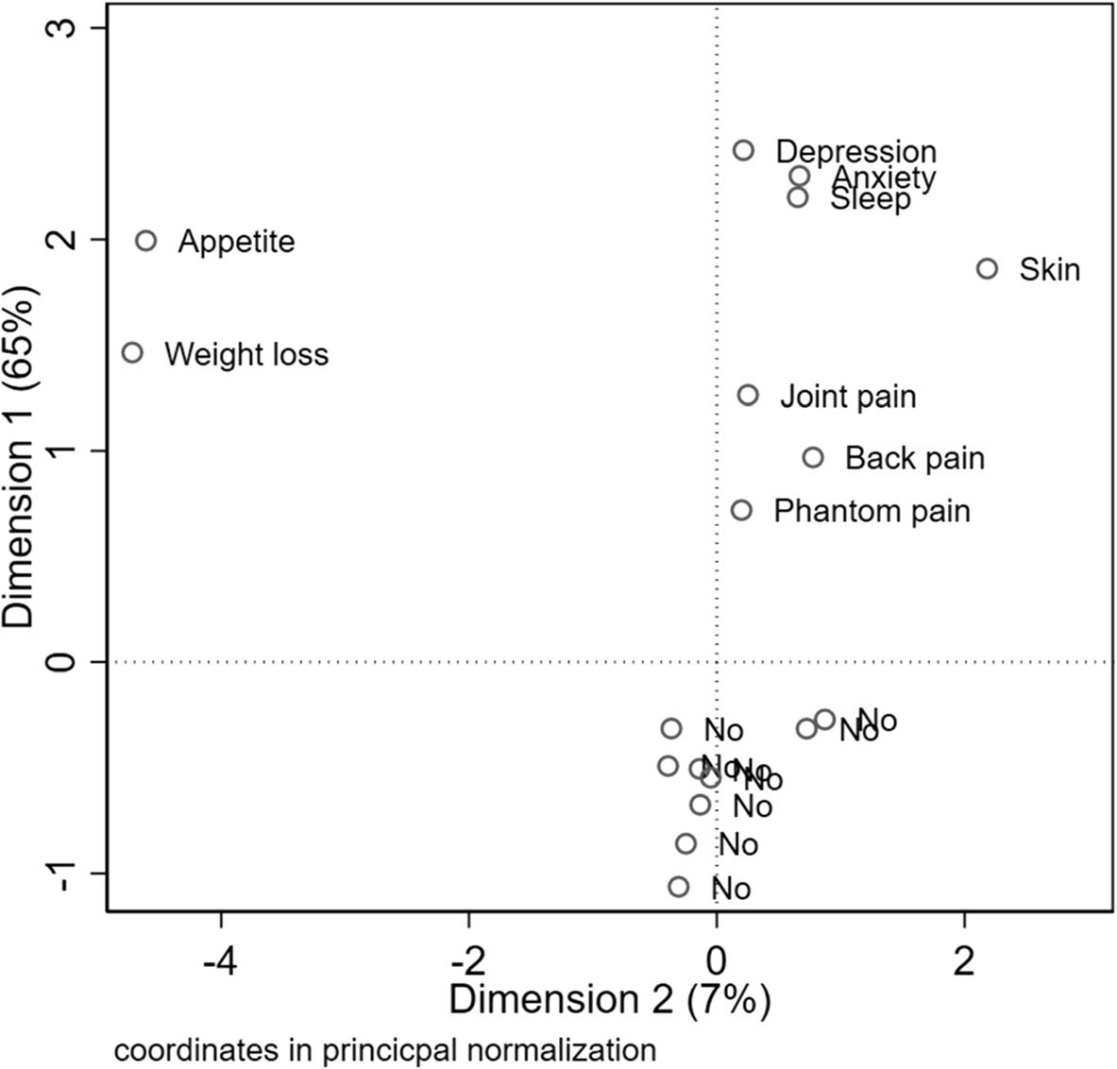
Relationships between long-term complications and frequencies (*N* = 254). *N = 254. The alluvial flow diagram shows how the general category (lower limb and upper limb left side) contains parts of finer classification, moving towards the right. The bold vertical lines indicate relative frequency. The colour code shows how each category moves into sub-categories, but change colour in the next step

The amputees reported physical and psychological problems in relation to the limb loss. Pain was most common, typically phantom pain (103/245, 40·6%), back pain (84/254, 33·1%) and joint pain (87/254, 34·3%). The dominating psychological problem were insomnia (69/254, 27·2%), anxiety (79/254, 31·1%) and feeling depressed (46/254, 18·1%) (Table 5.) By using multiple correspondence analysis (MCA), we found that some complications occurred more frequently in conjunction. Joint-, back and phantom pain occurred frequently together, as did depression, anxiety and sleep disturbances (Fig. 4).

**Table 5:** Medical and psychological complications

Pain	n	%	Mechanical	n	%	Systemic	n	%
Joint	87	34	Knee arthritis^b^	10	4	Weight loss	39	15
Back	84	33	Hip arthritis	2	0·9	Anorexia	34	13
Heel	21	8	Ankle arthritis	2	0·9	Insomnia	69	27
Phantom^a^	103	41	Knee stiffness	24	9	Depression^c^	46	18
			Hip stiffness	1	0·5	Anxiety^d^	79	31
			Ankle stiffness	9	4			
			Plantar fascitis	5	2			

Number of participants: 254, from 0 to 2% of the participants had missing data on any variable

^a^Phantom pain, defined as pain occurring in the amputated limb

^b^Stiffness refers to self-reported stiffness in joints, arthritis is self-reported as previous known diagnosis

^c^Depression refers to “feeling depressed”

^d^Anxiety refers to “feeling anxious”

## Discussion

In this study of 254 Gaza Palestinians who had sustained war-related traumatic extremity amputations and were currently attending physical rehabilitation, nine out of ten had major amputations (85%) with unilateral lower extremity amputations as the most common type (64·5%). Nearly one in five amputees was a child. The majority of the amputees were young men, mostly family breadwinners. Close to half of the patients reported being unemployed because of their physical disabilities adding to the already exceptionally high unemployment rate in Gaza. Loss of a steady income may cause new burdens to the patients and their families in addition to the loss of limb(s).

The majority of the studied patients had suffered severe amputations affecting the lower extremities. Nearly one in five had bilateral lower leg amputations. In addition, one in five needed more than ten surgical operations after the initial trauma, illustrating the complexity of the injuries and challenges to the local medical system. In total, this patient population underwent around 1300 surgical operations following the trauma, adding burden to an already exhausted health care system [10]. In addition, the 254 included patients in this study represented a 28% increase in the total caseload of the ALPC proving the serious impact war-related amputations have on both health care (emergency and later surgeries) as well as the physical and psychosocial rehabilitation systems in Gaza.

One in three amputations (35%) were above the knee and 59% of these patients were using artificial limbs, compared to 72% of those amputated below the knee. Artificial limbs are more difficult to fit for above knee amputees [15].

Traumatic losses of limbs following military attacks can lead to long-lasting physical incapacitation as well as medical problems and complications [16]. The main medical and mental burdens among the Palestinians we studied were pain, depression, anxiety and insomnia. The dominating types of pain were phantom pain, joint- and back-pain. The amputees also reported weight loss and loss of appetite. This is in accordance with studies showing a high prevalence of anxiety and depression in traumatic amputees [17]. Phantom pain and skin problems are also common complications in trauma related amputees [18].

Our finding of a clear male predominance among traumatic amputees is concomitant with other studies on war-related amputations [19]. This pattern of a male predominance is also found in the United Nations’ documentation of major trends in fatalities among Palestinians and Israelis since the beginning of the second Intifada (Sept. 2000 - July 2007) [20]. Men constituted the majority of the killed among both Israelis (69%) and Palestinians (94%). Similar numbers were found among children with 87% boys among Palestinian children killed, 13% girls [20]. Among intensive care patients in Gaza’s main trauma hospital during “Operation Protective Edge” in 2014, 77·9% were male, and 74% of patients admitted to this hospital during “Operation Pillar of Defense” in 2012 were also males [21, 22]. Possible reasons for the male predominance among amputees were discussed with local health care personnel. More systematic research will be necessary to reach firm conclusions. Health care professionals in Gaza point out that gender role in Gaza will typically assign different obligations to males and females. The women tend to stay at home to look after children and elderly in times of food scarcity and limited security. The male members of the family are more obliged to leave the relative safety of the home to find food in the market. Further, males also take higher risks in terms of volunteering to arrange evacuation of wounded to the hospital, in helping relatives, neighbors and friends during ongoing attacks and during the recovery phase when a building has collapsed following bombardment. Females typically stay in the home during such hostilities and are expected to take less personal risk.

However, the societal impact of a male predominance should not be underestimated. More than half of the male participants in our study were the sole breadwinners of their families and 63% of the male amputees were economical responsible for more than three persons in their household. Gaza reached a 43·6% unemployment rate in 2017, with a youth unemployment at 58% [23]. Young graduates reached an unemployment of 53% during the first quarter of 2017 [24].

Long lasting poverty for patients and families are known to be severe secondary trauma contributing to pain, insomnia and depression [25]. We have documented an unemployment rate at 75% among the amputees, extending the harm of war trauma, both for the amputees and their families [9]. Previous studies from Gaza found females in the region to feel less secure, and related this to the potential loss of the male breadwinner in the family [26].

Lacking a safe family housing may inflict severe strain on war injured patients, adding to unemployment. Almost half of the study population lost their home in one of the military incursions. Few homes had been rebuilt at the time of the study (11·6%, *n* = 26). Around 4000 families including 23.500 individuals were still displaced at the end of November 2017 following the military incursion, Operation Protective Edge, in 2014 [27]. To witness the destruction of your own home by enemy soldiers, as many of the amputees had done, is a significant psychological trauma [28].

The patients interviewed and examined after operation Protective Edge in 2014, shared in-depth stories about family members they had lost. Nine of the amputees in the study had lost one or several children in the conflict. This further illustrate the extent of profound, personal losses traumatic amputees in Gaza have suffered beyond the mere loss of limb(s).

Children injured or killed composed a considerable part of the civilian casualties in 2014 (556 killed, approx. 3.500 injured) [29].

### Limitations and strengths

The use of self-reported reasons for unemployment is a potential weakness of our study, though self-reported data are generally accurate [30]. The most recent cases were in an early stage of treatment and rehabilitation and would probably be more unlikely to be able to work, this may be a weakness in the self-reported data in the study. There is a potential for selection bias is present in our study. Some amputees sustain fatal injuries while others with minor injuries may not be referred for rehabilitation. Thus, although treatment at ALPC is free of charge, the patients attending ALPC are not representative of the whole population of amputees, as our study population does not include those who died before rehabilitation, or who did not need rehabilitation. However, due to the ongoing conflict, the lack of infrastructure and sometimes incomplete medical records, it would be close to impossible to obtain a completely representative set of data from the whole population of amputees.

Young men constituted the majority of patients in the study, and could indicate that they might have had a non-civilian status in the conflict. Ethical and security concerns limited the validation of social status beyond the information given by each amputee. Such information in the registry could pose a risk to them. We relied on the oral information obtained from each patient and local medical staff in our research group. Based on this, we are confident that more than 90% of the study participants were civilians.

Strengths of this study include a representative sample of the patients attending rehabilitation at ALPC (response rate 99%).

## Conclusions

In summary, amputation trauma following Israeli military incursions on the Gaza Strip is a major health problem for the otherwise healthy young civilians in our study, in particular for young adult men and for children. In addition to their amputation trauma, they suffer destruction of their homes, loss of family members and friends as well as significant financial deterioration.

These preventable, man-made traumatic amputations add significant physical, medical and psychological burdens to a civilian population who has also endured more than ten years of siege and blockade.

## Footnotes

1 The first author was contacted by email and permission to use their questionnaire was given in writing.
